# COVID-19 Vaccine Attitude and Its Predictors Among People Living With Chronic Health Conditions in Ibadan, Nigeria

**DOI:** 10.3389/ijph.2022.1604811

**Published:** 2022-10-14

**Authors:** Lucia Yetunde Ojewale, Rotimi Felix Afolabi, Adesola Ogunniyi

**Affiliations:** ^1^ Department of Nursing, Faculty of Clinical Sciences, College of Medicine, University of Ibadan, Ibadan, Nigeria; ^2^ Department of Epidemiology and Medical Statistics, Faculty of Public Health, College of Medicine, University of Ibadan, Ibadan, Nigeria; ^3^ Department of Medicine, Faculty of Medicine, College of Medicine, University of Ibadan, Ibadan, Nigeria

**Keywords:** Nigeria, COVID-19 vaccine, chronic disease, vaccination attitude, vaccine uptake, positive attitude, chronic health conditions

## Abstract

**Objective:** To assess vaccination attitude and its associated factors among people with chronic health conditions.

**Methods:** In this cross-sectional study, participants were 423 patients with chronic medical conditions. Data were collected on socio-demographic and COVID-19-related characteristics, *via* Open Data Kit software. A Vaccination Attitudes Examination (VAX) Scale was adopted. The main outcome was vaccine attitude status defined as positive if a VAX sum score was above the median value; otherwise, non-positive. Data were analysed using Chi-square and multivariate logistic regression analyses, at 5% level of significance.

**Results:** Overall proportion of patients with a positive attitude towards COVID-19 vaccination uptake was 46.6%. The most influential factor towards positive attitude was rating the government high in handling the pandemic. Other factors were education, income, COVID-19 knowledge and living room arrangement (*p* < 0.05).

**Conclusion:** Less than half of people living with a chronic medical condition had a positive attitude towards the COVID-19 vaccine. The attitudes are strongly mediated by confidence in the government. The government could promote a positive vaccine attitude by improving the clarity of health instructions that shows government transparency and effective communication. These are critical tools for maintaining public trust and confidence.

## Introduction

The impact of the COVID-19 pandemic on social, economic and political life is unprecedented. Lockdown was imposed in several countries to guarantee limited contact between individuals and to ensure that citizens observed social distancing, which admissibly helped curtail virus transmission [1]. However, countries suffered massive economic losses [2]. This led to easing the lockdown which soon increased COVID-19 infection and mortality [3]. The health system has not been able to effectively cater for the needs of those suffering from Acute Respiratory Distress (ARD) and SARS-CoV-2 cases of pneumonia, especially in Africa [4]. Consequently, efforts to prevent further transmission through the development of vaccines became inevitable. Vaccine development was seen as crucial to ending the pandemic [5]. As of late February 2021, COVID-19 vaccines were already available and were being administered to people mostly in high-income countries like the United Kingdom, United States, Canada, and China, among others [6]. In line with this and the effort by the World Health Organisation to ensure that the vaccines get to low- and middle-income countries, there is a need to ensure that it is well received by the general population, particularly those with chronic conditions.

Nonetheless, vaccine availability does not necessarily translate to the uptake. It has been suggested that apart from prioritizing vaccine administration, other important factors that would affect vaccine distribution include the capability of the health system in ensuring that the vaccines are made available for people at high risk and the willingness of the people to be vaccinated [7]. Despite the fatality of COVID-19 and the purported success in developing vaccines, a sceptical attitude continues to trail vaccination in many countries of the world, including developed countries. This phenomenon sometimes referred to as ‘vaccine hesitancy, has been reported in many countries largely due to vaccine disinformation [8, 9] and misinformation [10]. Latkin et al. (2021) reported that 40.9% of adults in the US mistrusted the vaccine while 16% of adults in the UK had a high level of mistrust, towards the vaccine [11]. Also, 69% of adult participants were willing to get vaccinated in a study among over 2000 adults in the US [12]. The proportion of people intending to get vaccinated is similar to that reported among about a thousand Hong Kong nurses [1]. Other authors have reported varying percentages in different African countries. These include 59% among Ethiopian health practitioners [13]; 44% and 51% among the general population of adults in Egypt and Ghana respectively [14, 15].

It was opined that the proportion of vaccinated people should be greater than two-thirds to achieve “herd” or public immunity [1]. Literature has shown that reasons for unwillingness to vaccinate, mistrust of vaccines and poor vaccine intention include low socioeconomic status, lower education, older age, concerns about the unforeseen side effects, mistrust of Government, poor adherence to COVID-19 prevention guidelines, being male, being unmarried, religious beliefs and social media influence [1, 5, 10, 11, 13, 15].

Addressing the aforementioned factors may promote a positive attitude and high intention to be vaccinated. Besides, COVID-19 vaccine recommendation by health care providers was a positive mediating factor towards its uptake among participants in several low- and middle-income countries [16] and among US adults [12]. Adequate understanding of clients by health care professionals is therefore crucial before they can effectively communicate the need for vaccines [17]. Nurses, physicians and other health care workers can thus effectively improve the attitude and uptake of the vaccine among their clients.

Generally, the population at higher risk of death or complications should be vaccinated first due to an inadequate supply of COVID-19 vaccine. Many countries have therefore adopted the WHO vaccination guideline of prioritizing people at higher risk of mortality from the disease including older adults and those with chronic conditions like diabetes and chronic kidney disease [18].

Specifically, adults with a chronic condition are more likely to be hospitalized due to COVID-19 infection compared to healthy individuals in a study conducted in the United States [19]. For example, having diabetes increases the risk of developing COVID-19 as well as increasing the risk of dying from COVID-19 complications [20]. Studies in Africa including Nigeria have also corroborated the claim that people who have co-morbidities such as diabetes and hypertension are more likely to suffer fatality from COVID-19 infection [21].

In a study conducted among Ethiopians living with diabetes and hypertension, many participants (79%) felt that they were more susceptible to COVID-19 death, yet only 10% were involved in a good level of COVID-19 prevention measures [22]. The poor attitude towards COVID-19 prevention could be carried over to the reception of the vaccine. Hence, the need to assess vaccination attitude.

Many studies have examined the COVID-19 vaccination attitude but few were conducted in Nigeria among people with chronic conditions. The attitude towards vaccination and its associated factors may differ considerably in Nigeria. While most studies have focused on the general population [9, 11, 12, 23] others have examined the phenomenon among specific populations including nurses [1] and students [24]. But there is a need to ascertain the attitude of people living with chronic conditions including diabetes, hypertension and chronic kidney disease towards vaccination since they are among the COVID-19 infection susceptible population for the uptake of the COVID-19 vaccine. . Against this background, the study aimed at assessing the COVID-19 vaccine attitude among persons living with chronic health conditions, receiving treatment in University College Hospital Ibadan, Nigeria, and to determine its associated factors.

## Methods

### The Study Design and Setting

A cross-sectional study on attitude and intention to COVID-19 vaccine uptake among people living with chronic health conditions in Ibadan, Nigeria was conducted between March and April 2021. This study is part of a larger study on “COVID-19 Vaccine: Attitude, Intention to Vaccinate, Mediating Factors and Interventions towards a Positive Attitudes among People with Chronic Conditions in Ibadan”. The present study was conducted at the Medical Outpatient Clinic of the University College Hospital (UCH), Ibadan, Oyo State, Nigeria. The UCH is a tertiary health care facility which specialises in all disease conditions and receives referrals from secondary health facilities within and outside Oyo State. Eligible consenting patients were referred to participate by Counselors after routine patients’ education sessions during which information about the study was provided to all clinic attendees. On average, 20 participants were expected on each clinic day for the 2 months of data collection.

### Sample Size Determination and Sampling Strategy

Based on the assumptions of a 50% prevalence of positive attitude towards the uptake of COVID-19 vaccination among the patients and a 5% desired level of precision, the required minimum sample size was estimated. The assumed 50% prevalence was adopted since no other studies had been conducted in Nigeria, as of February 2021 when the study protocol was developed, to show the composite level of attitude to COVID-19 vaccine uptake. A total of 423 sample size was then estimated for the study after adjusting for a 10% non-response rate. At every clinic visit, eligible consenting participants were selected using a simple random sampling (balloting approach), conducted by researchers. Daily attendance register at the Record section of the clinic served as the sampling frame. Of 40 secret ballot papers, labelled “yes” or “no” prepared for eligible patients who registered on a clinic day, 20 were “yes.” A patient who selected a “yes” was enrolled on the study after written informed consent was obtained while excluding patient who was very ill and cognitively impaired.

### Data Collection

Data collection took place before the vaccination of the general population commenced in Nigeria. Data were collected on socio-demographic variables and COVID-19-related characteristics among the patients by trained research assistants. The interviewers who were postgraduate students in the College of Medicine were trained at a one-day workshop. During the training, they got general orientation about the study objectives, interviewing skills and health research ethics. Each question item was explained as well as how to record the responses.

A questionnaire consisting of three [3] sections was used for data collection. The first section consisted of sociodemographic data and predictors of vaccination attitude, based on a literature search. Items included were gender, age, socioeconomic status using the wealth index [25], employment classification, history of children’s vaccination, daily exposure to news, and self-rated adherence to the COVID-19 guidelines, among others.

The second section was made up of the Vaccine Attitude Examination (VAX) Scale. The VAX scale is a 12-item scale [26]. The scale consisted of four subscales which provide information on individuals with vaccination resistance. The subscales are 1) mistrust of vaccine benefits 2), worries about unforeseen future effects 3), concerns about commercial profiteering, and 4) preference for natural immunity. A sufficient convergent validity and internal reliability (Cronbach’s alphas = 0.77–0.93) had been established for all four subscales [26]. The scale is rated on a six-point Likert scale (very strongly disagree (coded as 0), strongly disagree (coded as 1), disagree (coded as 2), agree (coded as 3), strongly agree (coded as 4) and very strongly agree (coded as 5). With a maximum possible score of 60, the overall score was dichotomized using the median value as a cut-off value.

The last section was made up of questions to ascertain contextual influences on COVID-19 vaccine attitude. It was made up of 16 items with three main options: “Yes/No/Not sure.”.

### Data Processing and Analysis

The analysis started with data cleaning to ensure completeness and consistency. The main outcome variable was attitude towards the uptake of the COVID-19 vaccine. The response to the attitude questions was summed together to generate an attitude score ranging from 0 to 60. Similar scores (ranged 0–15 scores) were generated for each of the four subscales of attitude toward COVID-19 vaccine uptake. Having confirmed the nonnormality of the outcome variable including its subscales’ scores using the Shapiro Wilk normality test (*p* < 0.05), an overall score above the median value was coded “1” as a positive attitude; otherwise, coded “0” as non-positive [27]. Independent variables considered were socio-demographics, contextual and COVID-19-related characteristics, see Table 1.

**TABLE 1:** Definitions of independent variables (Nigeria, 2021).

Characteristics	Description
Age	Patient’s age (in years) categorised as: <30, 30–49, 50–64, ≥65 years
Sex	Sex of the patient (male or female)
Ethnicity	Patient ethnicity (Yoruba, others)
Religion	The religion of patients is grouped into two: Christianity, Islam
Marital	Patient marital status: Never married, married
Employment	Patient employment status: skilled, unskilled
Education	Patient’s highest level of educational attainment categorised as no formal, primary, secondary, higher
Income	Patient monthly income (in Naira) categorised as: <30,000, 30,000–49,999, 50,000–99,999, ≥100,000, unknown
Assets owned	Patient total assets grouped into single (only one asset declared) or multiple (two or more assets declared)
Media exposure	Exposure to media is grouped into not exposed and exposed
Cooking fuel	Type of cooking fuel categorised as clean and unclean
Drinking water source	Source of drinking water grouped as improved and unimproved
Toilet facility	Household toilet facilities: improved; unimproved
Waste disposal	Waste disposal practice: hygienic; unhygienic
No of rooms	Residential house number of rooms categorised as 1–2, 3–4, >4
No of persons	Number of persons living in a house categorised as 1–2, 3–4, 5–6, >6
Health condition	Patient health challenges are grouped as single or multiple diseases
COVID-19 knowledge	Rated level of COVID-19 knowledge grouped into poor and good
Adherence level	Adherence to the COVID-19 guideline: poor, better
Full child vaccination	Participant’s children were full vaccination: complete and incomplete/none
Conf in govt	Patient rated level of confidence in government in handling pandemic: undecided, low, high
Conf in health prof	Patient-rated level of confidence in health care professionals: undecided, low, high

Descriptive statistics such as percentages were used to report the frequency distribution and prevalence of the overall positive attitude towards the uptake of the COVID-19 vaccine, including its four subscales, by the independent characteristics. Chi-squared and Fisher exact tests (where applicable) were performed to assess the individual association of selected background characteristics with the positive attitude towards COVID-19 vaccine uptake in each of the subscales. All factors significantly (*p* < 0.25) associated with a positive attitude towards COVID-19 vaccination at the bivariate level were thereafter included at the multivariate stage. The logistic regression was used to determine the influence of selected background characteristics on the positive attitude towards COVID-19 vaccine uptake. The adjusted odds ratios (aORs) including their 95% confidence intervals (CIs) and/or *p*-values are reported. Data management and analysis were conducted using Stata version 14.0 statistical software at a 5% level of significance.

### Ethical Approval

The University of Ibadan/University College Hospital Institutional Review Committee approved the survey protocol with approval number UI/EC/21/0065. Participants gave informed consent and were briefed on their freedom to withdraw from the interview at any point, before data collection. Every tenet of the Helsinki declaration and other ethical requirements were strictly complied with throughout the study. No identifying information was collected from participants and study questionnaires were accessible to only investigators and authorised research staff.

## Results

### Participants’ Characteristics

The participants’ mean age was 54.3 (standard deviation [SD]: 16.3) years. Most participants were aged 50–64 years (35.7%), women (58.2%) and Yoruba (91.3%). Only about 8.0% of respondents earned less than the national minimum wage and 8.5% had no formal education. Most participants reported single health conditions (88.7%) (Table 2).

**TABLE 2:** Socio-demographic and contextual characteristics of the participants (Nigeria, 2021).

Characteristics	n	%
Age (years)		
<30	36	8.5
30–49	112	26.5
50–64	151	35.7
≥65	124	29.3
Sex		
Male	177	41.8
Female	246	58.2
Ethnicity		
Yoruba	386	91.3
Other	37	8.7
Religion		
Christianity	292	69
Islam	131	31
Marital status		
Not married	48	11.3
Married	375	88.7
Employment status		
Skilled	313	74
Unskilled	110	26
Highest education		
No formal	36	8.5
Primary	87	20.6
Secondary	130	30.7
Higher	170	40.2
Income (monthly)		
<₦30,000	34	8
₦30,000 – ₦49,999	72	17
₦50,000 – ₦99,999	92	21.8
≥₦100,000	55	13
Unknown	170	40.2
Asset own		
Single	307	72.6
Multiple	116	27.4
Media exposure		
Not Exposed	32	7.6
Exposed	391	92.4
Cooking fuel		
Clean	17	4
Unclean	406	96
Drinking water source		
Improved	219	51.8
Unimproved	204	48.2
Toilet facility		
Improved	402	95
Unimproved	21	5
Waste disposal		
Hygienic	176	41.6
Unhygienic	247	58.4
No of rooms		
1–2	109	25.8
3–4	213	50.4
>4	101	23.9
No of persons		
1–2	96	22.7
3–4	142	33.6
5–6	156	36.9
>6	29	6.9
Health condition		
Single	375	88.7
Multiple	48	11.3

n, number of subjects per group.

**FIGURE 1:**
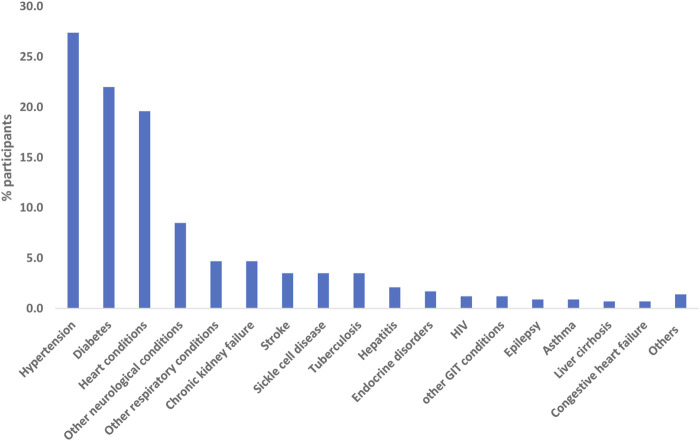
Percentage distribution of participants’ chronic health conditions [multiple responses] (Nigeria, 2021).

Hypertension (*n* = 116; 27.4%), diabetes mellitus (*n* = 93; 22.0%) and heart conditions/diseases (*n* = 83; 19.6%) were the top three conditions (see Figure 1).

As shown in Table 3, a majority (84.9%) of the participants rated their COVID-19 knowledge high while several (59.3%) had poor adherence to the COVID-19 prevention protocol. Just over half (52.0%) had high confidence in the government while a greater percentage had high confidence in health care workers (79.7%).

**TABLE 3:** COVID-19-related characteristics of the participants (Nigeria, 2021).

Characteristics	n	%
COVID-19 knowledge		
Poor	64	15.1
Good	359	84.9
Adherence level		
Poor	251	59.3
Better	172	40.7
Full child vaccination		
Incomplete	84	19.9
Complete	339	80.1
Conf. in govt		
Low	143	33.8
Undecided	60	14.2
High	220	52
Conf. in health prof		
Low	42	9.9
Undecided	44	10.4
High	337	79.7

n, number of subjects per group.

Table 4 shows that the proportion of positive attitudes towards COVID-19 vaccine uptake increased with increasing respondents’ age and levels of confidence in government handling the pandemic, in each of the subscales. Overall, less than half of the respondents (46.6%) had a positive attitude towards the uptake of the COVID-19 vaccine. Almost an equivalent proportion of participants had a positive attitude relating to COVID-19 vaccination against worries about unforeseen future effects (46.6%) and preferences for natural immunity (45.9%); 29.6% against mistrust of vaccine benefits and 11.1% were concerned about commercial profiteering.

**TABLE 4:** Distribution of participants and proportion of positive attitude towards COVID-19 vaccine according to selected characteristics by attitude subscales (Nigeria, 2021).

	Attitude subscale									
	All		Mistrust		Worries		Concern		Preference	
Characteristics	% +ve	p^	% +ve	p^	% +ve	p^	% +ve	p^	% +ve	p^
Age (years)		0.315		0.197		0.146		0.675		0.037*
<30	36.1		13.9		38.9		5.6		63.9	
30–49	42.9		31.3		45.5		11.6		37.5	
50–64	47.7		30.5		42.4		12.6		45	
≥65	51.6		31.5		54.8		10.5		49.2	
Sex		0.498		0.310		0.777		0.464		0.106
Male	44.6		32.2		45.8		12.4		41.2	
Female	48		27.6		47.2		10.2		49.2	
Ethnicity		0.032*		0.268		0.441		0.784		0.305
Yoruba	48.2		30.3		47.2		11.4		46.6	
Other	29.7		21.6		40.5		8.1		37.8	
Religion		0.092		0.147		0.400		0.882		0.820
Christianity	43.8		27.4		45.2		11		46.2	
Islam	52.7		34.4		49.6		11.5		45	
Marital status		0.181		0.784		0.677		0.225		0.220
Not married	37.5		31.3		43.8		6.3		54.2	
Married	47.7		29.3		46.9		11.7		44.8	
Employment status		0.785		0.543		0.538		0.938		0.730
Skilled	47		30.4		45.7		11.2		45.4	
Unskilled	45.5		27.3		49.1		10.9		47.3	
Highest education		0.006*		0.382		0.003*		0.939		0.031*
No formal	72.2		19.4		75		13.9		69.4	
Primary	48.3		29.9		41.4		10.3		44.8	
Secondary	46.9		33.9		47.7		11.5		43.1	
Higher	40		28.2		42.4		10.6		43.5	
Income (monthly)		0.015*		0.155		0.276		0.052		0.000*
<₦30,000	61.8		14.7		58.8		8.8		79.4	
₦30,000–₦49,999	59.7		38.9		52.8		16.7		40.3	
₦50,000–₦99,999	44.6		29.4		43.5		7.6		48.9	
≥₦100,000	34.6		29.1		38.2		20		10.9	
Unknown	42.9		28.8		45.9		8.2		51.2	
Asset own		0.049		0.010*		0.823		0.034*		0.000*
Single	49.5		26.1		46.9		9.1		54.4	
Multiple	38.8		38.8		45.7		16.4		23.3	
Media exposure		0.011		0.854		0.285		0.795		0.536
Not Exposed	25		28.1		37.5		12.5		40.6	
Exposed	48.3		29.7		47.3		11		46.3	
Cooking fuel		0.301		0.579		0.649		0.237		0.037
Clean	58.8		23.5		41.2		0.0		70.6	
Unclean	46.1		29.8		46.8		11.6		44.8	
Drinking water source		0.011		0.223		0.329		0.099		0.000*
Improved	52.5		26.9		48.9		8.7		54.8	
Unimproved	40.2		32.4		44.1		13.7		36.3	
Toilet facility		0.148		0.279		0.319		0.493		0.050
Improved	45.8		30.1		46		11.4		44.8	
Unimproved	61.9		19.1		57.1		4.8		66.7	
Waste disposal		0.000*		0.031		0.326		0.087		0.000*
Hygienic	36.4		35.2		43.8		14.2		27.8	
Unhygienic	53.9		25.5		48.6		8.9		58.7	
No of rooms		0.009*		0.023		0.204		0.000*		0.000*
1–2	33.9		32.1		41.3		10.1		43.1	
3–4	51.6		23.9		46		6.1		58.7	
>4	49.5		38.6		53.5		22.8		21.8	
No of persons		0.716		0.824		0.492		0.881		0.004*
1–2	47.9		29.2		52.1		12.5		49	
3–4	45.1		32.4		47.2		12		38.7	
5–6	48.7		27.6		44.2		9.6		54.5	
>6	37.9		27.6		37.9		10.3		24.1	
Health condition		0.677		0.542		0.181		0.515		0.997
Single	46.9		29.1		47.7		11.5		45.9	
Multiple	43.8		33.3		37.5		8.3		45.8	
COVID-19 knowledge		0.745		0.019*		0.623		0.004*		0.000*
Poor	48.4		17.2		43.8		1.6		70.3	
Good	46.2		31.8		47.1		12.8		41.5	
Adherence level		0.000*		0.076		0.226		0.002*		0.000*
Poor	53.8		26.3		49		7.2		59.8	
Better	36.1		34.3		43		16.9		25.6	
Full child vaccination		0.082		0.198		0.784		0.039*		0.020*
Incomplete	38.1		23.8		45.2		4.8		57.1	
Complete	48.7		31.0		46.9		12.7		43.1	
Conf. in govt		0.000*		0.000*		0.000*		0.000*		0.000*
Low	16.8		16.1		26.6		2.1		39.2	
undecided	23.3		13.3		30		5		28.3	
High	72.3		42.7		64.1		18.6		55	
Conf. in health prof		0.000*		0.009*		0.001*		0.270		0.398
Low	26.2		19.1		21.4		4.8		45.2	
undecided	29.6		13.6		40.9		6.8		36.4	
High	51.3		32.9		50.5		12.5		47.2	
Total	46.6		29.6		46.6		11.1		45.9	

*Significant at 5%; n – number of subjects per group; +ve – positive attitude; ^ *p*-value at 5% chi-square or Fisher exact test of association.

### Factors Influencing Positive Attitude Towards COVID-19 Vaccination Among People Living With Chronic Health Conditions

The adjusted associations of positive attitude towards COVID-19 uptake with significant characteristics (*p* < 0.25) at the bivariate level, including the attitude subscales set out in Models 1–4, are presented in Table 5. In the overall model, the likelihood of having a positive attitude towards COVID-19 vaccine uptake was higher among patients living in a house with more than two rooms (3-4 rooms—aOR = 3.16, CI: 1.52, 6.57; >4 rooms—aOR = 4.29, CI: 1.87, 9.87) and those who rated government high (aOR = 15.78, CI:6.52, 38.16) at handling the pandemic. The odds of a positive attitude was decreased among patients who had primary education (aOR = 0.28; CI: 0.10, 0.84) and those who had a high level of adherence to COVID-19 preventive measures (aOR = 0.50; CI: 0.29, 0.86). In all the models, patients who rated the government’s handling of the pandemic high were more likely to have a positive attitude toward COVID-19 vaccination. Additionally, patients who owned multiple assets were more likely to have a trust of vaccine benefits (see, model 1). Patients who had formal education were more worried about unforeseen future effects of the COVID-19 vaccine. Residents of a house with more than four rooms were less worried about unforeseen future effects (see, model 2). Living in a house of more than four rooms significantly lowered concerns about commercial profiteering (see, model 3). Patients who practised unhygienic waste disposal (aOR = 2.17; CI:1.25, 3.77) had lower odds of preferences for natural immunity. The odds of preferences for natural immunity were increased among patients who earned more than #30,000 and among those who had better adherence to COVID-19 preventive measures (aOR = 0.44; CI: 0.26, 0.73) (see, model 4, Table 5).

**TABLE 5:** Adjusted odds ratios positive attitude towards COVID-19 vaccine uptake (Nigeria, 2021).

Background characteristics	Overall attitude	Attitude subscales: aOR (95% CI)			
	Or (95%CI)	Model 1 (Mistrust)	Model 2 (Worries)	Model 3 (Concern)	Model 4 (Preference)
Age (years)					
<30		1	1		1
30–49		2.98 (0.91, 9.72)	1.28 (0.55, 2.97)		0.45 (0.13, 1.61)
50–64		2.40 (0.75, 7.69)	0.83 (0.36, 1.92)		0.65 (0.18, 2.38)
≥65		2.34 (0.70, 7.77)	1.53 (0.62, 3.72)		0.72 (0.19, 2.75)
Sex					
Male					1
Female					0.99 (0.58, 1.67)
Ethnicity					
Yoruba	1.45 (0.50, 4.16)				
Other	1				
Religion					
Christianity	0.92 (0.50, 1.66)	0.83 (0.48, 1.43)			
Islam	1	1			
Marital status					
Not married	1			1	1
Married	0.86 (0.33, 2.23)			0.79 (0.21, 2.98)	1.43 (0.48, 4.30)
Highest education					
No formal	1		1		1
Primary	0.28 (0.10, 0.84)*		0.19 (0.08, 0.47)*		0.53 (0.18, 1.54)
Secondary	0.43 (0.16, 1.13)		0.34 (0.14, 0.84)*		0.61 (0.21, 1.77)
Higher	0.58 (0.19, 1.77)		0.30 (0.12, 0.74)*		1.57 (0.49, 4.99)
Income (monthly)					
<₦30,000	1	1		1	1
₦30,000 – ₦49,999	1.76 (0.51, 6.05)	3.23 (0.94, 11.09)		1.69 (0.23, 5.03)	0.29 (0.09, 0.89)*
₦50,000 – ₦99,999	0.53 (0.14, 1.98)	1.68 (0.49, 5.76)		0.33 (0.06, 1.41)	0.29 (0.09, 0.94)*
≥₦100,000	0.57 (0.12, 2.70)	1.10 (0.28, 4.36)		0.91 (0.14, 4.81)	0.06 (0.01, 0.29*)
Unknown	0.86 (0.27, 2.72)	2.10 (0.63, 6.98)		0.52 (0.10, 2.16)	0.43 (0.14, 1.27)
Asset own					
Single	1	1		1	1
Multiple	1.04 (0.54, 2.00)	1.87 (1.06, 3.30)*		1.43 (0.60, 3.36)	0.58 (0.30, 1.10)
Media exposure					
Not Exposed	1				
Exposed	1.31 (0.39, 4.34)				
Cooking fuel					
Clean					1.11 (0.27, 4.52)
Unclean					1
Drinking water source					
Improved	1	1		1	1
Unimproved	0.74 (0.42, 1.29)	1.23 (0.73, 2.09)		1.43 (0.63, 2.84)	0.84 (0.49, 1.43)
Toilet facility					
Improved	1				1
Unimproved	2 (0.46, 8.71)				1.08 (0.23, 5.01)
Waste disposal					
Hygienic	1	1		1	1
Unhygienic	1.42 (0.80, 2.52)	0.63 (0.36, 1.12)		0.82 (0.38, 1.80)	2.17 (1.25, 3.77)*
No of rooms					
1–2	1	1	1	1	1
3–4	3.16 (1.52, 6.57)*	0.75 (0.40, 1.41)	1.52 (0.87, 2.60)	0.64 (0.24, 1.69)	1.79 (0.89, 3.61)
>4	4.29 (1.87, 9.87)*	1.55 (0.77, 3.14)	2.38 (1.29, 4.40)*	2.96 (1.10, 8.68)*	0.55 (0.24, 1.29)
No of persons					
1–2					1
3–4					0.65 (0.32, 1.32)
5–6					1.35 (0.63, 2.91)
>6					0.73 (0.22, 2.41)
Health condition					
Single			1		
Multiple			0.59 (0.29, 1.17)		
COVID-19 knowledge					
Poor		1		1	1
Good		1.18 (0.51, 2.70)		3.85 (0.34, 35.85)	0.49 (0.23, 1.06)
Adherence level					
Less	1	1	1	1	1
Much	0.50 (0.29, 0.86)*	1.1 (0.65, 1.86)	0.84 (0.54, 1.31)	1.90 (0.89, 3.96)	0.44 (0.26, 0.73)*
Full child vaccination					
Incomplete	1	1			1
Complete	1.01 (0.43, 2.33)	0.8 (0.35, 1.79)			0.63 (0.29, 1.39)
Conf. in govt					
Low	0.80 (0.33, 1.95)	1.32 (0.48, 3.59)	1.21 (0.57, 2.60)	0.41 (0.07, 2.47)	1.29 (0.55, 3.04)
undecided	1	1	1	1	1
High	15.78 (6.52, 38.16)*	6.18 (2.44, 15.66)*	5.84 (2.77, 12.32)*	7.09 (1.82, 30.24)*	3.6 (1.58, 8.19)*
Conf. in health prof					
Low	0.92 (0.27, 3.11)	1.79 (0.44, 7.28)	0.35 (0.12, 1.06)		
undecided	1	1	1		
High	0.73 (0.30, 1.78)	1.53 (0.51, 4.58)	0.73 (0.35, 1.52)		
-LL	200.0	218.8	247.4	111.5	209.1
N	423	423	423	423	423

*Significant at 5%; + LL, log-likelihood; N, number of observations: Model 1 – modelled positive attitude against mistrust of vaccine benefit; Model 2 – modelled positive attitude against worries about unforeseen future effects; Model 3 – modelled positive attitude against concerns about commercial profiteering; Model 4 – modelled positive attitude against preference for natural immunity.

## Discussion

We determined COVID-19 vaccination attitude and the predictors among people living with chronic medical conditions in Ibadan, Nigeria. In Nigeria, researchers have assessed COVID-19 vaccine attitude among students [24], acceptance among the general populace [23, 28–30], hesitance among university community [31], perception and willingness to pay among community members [32] or willingness to uptake among health workers [33]. However, none has reported the vaccination attitude among people living with chronic conditions. This study, therefore, stands in the frontline to assess COVID-19 vaccine attitude and its determinants among patients living with chronic health conditions in Nigeria.

In this study, less than half of the participants had a positive attitude towards vaccine uptake. The relatively low level of positive attitude to vaccinate among the population group suggests vaccine hesitancy which may hinder herd immunity. Interestingly, the percentage reported is comparable to that reported among health workers and the general population in Nigeria [23, 33]. It was however higher than the percentage reported among staff and students of a tertiary institution in south-east Nigeria [31]. Compared to other African countries, the proportion reported in this study is lower than that reported in Ethiopia (59%) [34] and comparable to that of Egypt (44%) [14] and Ghana (51%) [15]; however, it was higher than the percentage reported in Kuwait (24%) [35]. In contrast, some studies have shown that participants in high-income countries had a higher percentage of positive attitudes toward vaccine uptake [11, 36]. This could be associated with a greater trust in the vaccine because of the local production of vaccines in those countries. Another plausible reason for the low percentage of positive attitudes towards the vaccine among the studied participants could be linked to the fear of side effects [24]. Fear of vaccine side-effects may therefore be an important predictor of vaccine uptake. Hence, there is a need for aggressive advocacy interventions that offer information on the uptake of the vaccine’s safety and efficacy, especially among the population at high risk.

The most influential factor in having a positive attitude towards the COVID-19 vaccine among the study participants was trust in the government’s ability to handle the pandemic. Those who trusted the government were sixteen times more likely to take the vaccine. This element of trust in the government was much stronger than the influence of confidence in health professionals. Generally, many people think, justifiably so, that the government has a lot to do in helping to manage the pandemic. Not surprising, in many other countries including Nigeria, the government was responsible for imposing curfews, ensuring COVID-19 testing, establishing isolation centres and procuring vaccines for the people. In the USA, the influence of the government had been documented before the COVID-19 pandemic as a significant predictor of flu vaccination [37]. It has also been reported to impact the COVID-19 vaccination attitude among Ireland and UK citizens [38]. This finding corroborates a study conducted among US, Australian and UK citizens [39].

Trust in the government and its influence on vaccination attitude does appear to be a common phenomenon among people across the globe. This was the case in a large intercontinental study involving twenty-six adults [14]. Likewise, another study [40] reported the influence of mistrust of the government on vaccine attitude. There is currently no health condition that has generated so much media attention, controversy/strongly polarised opinion and political involvement as issues surrounding the COVID19 pandemic. Hence, the citizens look up to authorities such as the government to resolve doubts and provide guidelines for the people. This view was supported by Soares et al [41] in a study among the general Portuguese population. Park et al. [42] also reported the negative influence of mistrust in government on vaccination attitudes among South Korean adults. Association of low level of confidence in government handling the pandemic and low level of positive vaccine attitude may result in unwillingness to vaccinate or uptake refusal [11]. It is therefore a matter of necessity that the government and her agencies further strategies to continually gain confidence and build trust in her citizenry for effective uptake of the COVID-19 vaccine in Nigeria.

Socio-economic status based on having more than two rooms in the house was associated with an overall positive attitude towards the vaccine. Similarly, those who owned multiple assets had a positive attitude and were more likely to trust vaccine benefits. These results align with the findings of other studies [2, 11, 43]. Socio-economic status was however not associated with COVID-19 vaccination attitude among older adults in the UK [44]. On the other hand, people of lower socioeconomic status as shown by having fewer assets, lower income and unhygienic waste disposal, which is common among those who live in urban slums, preferred the vaccine to their natural immunity. It is possible that this group of people could not afford a good diet and felt that their immune system was not strong enough to withstand COVID-19 infection [45, 46].

Patients who adhered more to the COVID-19 preventive guidelines had a lowered positive attitude towards the COVID-19 vaccine, but they were more likely to prefer natural immunity to the vaccine. This could be associated with their perceived belief that they would not contract the infection by meticulously following preventive measures. Perception of high COVID-19 risk has been reported to be associated with willingness to take the vaccine among health workers in Nigeria [33]. Our result is in contrast with that of Paul et al. [11] who reported a high level of trust in the COVID-19 vaccine among adults in the UK and with high adherence to COVID-19 measures. By implication, non-positive attitudes towards vaccine may stimulate vaccine mistrust and vaccine hesitancy. These may consequently hinder the attainment of general population immunity. Hence, enlightenment campaigns should be aimed at addressing these concerns.

The study further showed that participants who did not complete their children’s immunization showed a preponderance for natural immunity. This, however, poses a question of how far a person’s natural immunity can protect against the COVID virus. This is an aspect that requires exploration to get to the root of vaccine hesitancy. Recently, The British Society for Immunology in collaboration with the UK Coronavirus Immunology Consortium (UK-CIC) has stated that vaccination against COVID-19 is likely to lead to a more effective and longer-lasting immunity than that prompted by natural infection with the virus. The vaccine is also said to be five times more protective against the virus compared to natural immunity following the infection [47]. Participants who rated their COVID-19 knowledge high were not likely to be concerned about commercial profiteering through the vaccine. This is very likely because many might have sought information about the infection including the vaccine and were convinced of the genuineness of the manufacturers. Hong et al. [48] reported the influence of COVID-19 vaccine knowledge on vaccine acceptance. In view of the present study, the effect of COVID-19 knowledge on vaccine attitude substantially changed after controlling for other covariates. This indicates that having a good knowledge of COVID-19 disease alone may not have contributed to the relatively low levels of mistrust of vaccine benefits and concerns about future unforeseen side effects among people living with chronic health conditions in Nigeria.

The study further showed that participants who did not complete their children’s immunization showed a preponderance for natural immunity. This, however, poses a question of how far a person’s natural immunity can protect against the COVID-19 virus. This is an aspect that requires exploration to get to the root of vaccine hesitancy. Recently, The British Society for Immunology in collaboration with the UK Coronavirus Immunology Consortium (UK-CIC) has stated that vaccination against COVID-19 is likely to lead to a more effective and longer-lasting immunity than that prompted by natural infection with the virus. The vaccine is also said to be five times more protective against the virus compared to natural immunity following the infection [47]. However, the association becomes nonsignificant when other covariates were controlled for.

### Conclusion

Less than half of people living with chronic conditions in Ibadan Southwest Nigeria had a positive attitude towards the COVID-19 vaccine, suggesting a relatively low level of potential vaccine uptake. Their attitude was largely influenced by confidence in the government’s ability to handle the pandemic. Other mediating factors toward positive vaccine attitude were high socioeconomic status, poor adherence to COVID-19 preventive measures and having had no formal education. The findings may contribute to developing a strategy for controlling the pandemic by addressing factors significantly affecting vaccination attitude. It is therefore important to design a holistic strategy to promote the uptake of the COVID-19 vaccine, especially among people living with chronic conditions and other high-risk populations. This should include, in the first place, the government’s effort to create and maintain the trust of the citizens. Other measures could include targeting people of low socioeconomic and literacy levels.

### Recommendations/Implication

A low level of a positive attitude toward COVID-19 vaccines suggests a substantial barrier to attaining the vaccination uptake and coverage needed for herd immunity. Interventions to promote a positive attitude among people living with chronic conditions should consider factors such as socio-economic status, level of adherence to COVID-19 preventive measures and educational attainment. More importantly, the development of strategies demonstrating government capability at handling the pandemic and at gaining the citizenry’s trust will be critical for effective coverage and uptake of COVID-19 vaccines.

### Limitations

Data were collected from a single study setting and the observed attitudes may be biased towards those who have a good health seeking behavior.

### Suggestions for Further Study

A qualitative study on the reasons for poor vaccine attitude.
